# Blind Nasotracheal Intubation in a Patient With Ankylosing Spondylitis and Severe Cervical Spine Deformities: A Case Report on Challenges and Approaches to Difficult Airway Management

**DOI:** 10.7759/cureus.73164

**Published:** 2024-11-06

**Authors:** Nebojsa Brezic, Strahinja Gligorevic, Jovana Stanisavljevic, Adi Hadzibegovic, Bojan Jovanovic

**Affiliations:** 1 Department of Anesthesiology, Resuscitation and Critical Care, University Clinical Center of Serbia, Belgrade, SRB

**Keywords:** ankylosing spondylitis, blind nasotracheal intubation, cervical spine deformities, difficult airway management, intubation challenges

## Abstract

Airway management in patients with advanced ankylosing spondylitis (AS) presents a unique challenge due to possible cervical spine deformities that restrict neck mobility and affect access to the airway. Traditional airway management strategies, such as direct laryngoscopy and even fiberoptic intubation, are often rendered ineffective due to these anatomical limitations. Furthermore, surgical options like tracheostomy can be infeasible in cases with significant neck deformities, necessitating alternative approaches. This case depicts a 48-year-old male with untreated AS who presented to the emergency department following a fall, resulting in unstable vertebral fractures and paraplegia. The patient’s severe cervical deformities posed significant challenges to airway management, and conventional airway management strategies, including fiberoptic intubation, were unsuccessful. Given the impracticality of tracheostomy due to the anatomical limitations, blind nasal intubation was successfully performed in a semi-recumbent position. This case underscores the complexities of airway management in AS patients with severe deformities. It highlights the importance of alternative intubation strategies, even blind nasotracheal intubation, when conventional methods fail due to anatomical constraints.

## Introduction

Ankylosing spondylitis (AS) is a chronic inflammatory disorder of the axial skeleton resulting in progressive spinal rigidity and deformities [1]. The global prevalence of AS varies significantly across regions, ranging from a low of 0.074% in Africa to a high of 0.319% in North America. The condition affects males more than three times as often as females, with onset typically occurring in the second and third decades of life [2]. As the disease progresses, especially in untreated or poorly managed cases, spinal deformities may develop, severely limiting mobility and significantly complicating airway management [3]. Cervical spine involvement in AS becomes more prevalent with increasing age and disease duration, particularly in more symptomatic and structurally severe cases. Approximately 70% of AS patients develop cervical spine involvement after 20 years of disease progression [4], with severe cases of chin-on-chest hyperkyphosis also reported [5]. Osteoporosis is prevalent among patients with AS and is linked to a higher risk of spinal fractures, particularly in those with prolonged disease duration and advanced spinal involvement [3]. However, recent data indicate that even after adjusting for age, gender, and the presence of osteoporosis, patients with AS still have an elevated risk of developing vertebral fractures [6]. These fractures, particularly in the cervical spine, carry a high risk of neurological compromise, with more than one fourth of cervical spine fractures complicating with spinal cord injury [3]. Cervical fractures in AS are often unstable, and urgent intervention is required to prevent permanent spinal cord damage [7]. 

Airway management in patients with AS presents significant challenges due to various anatomical limitations. These patients often have restricted atlanto-occipital extension and may be unable to lie supine due to spinal fusion and anterior flexion of the cervical spine [3]. Additionally, temporomandibular joint (TMJ) ankylosis in AS, occurring in up to 35% of patients, can lead to microstomia, further restricting mouth opening and complicating airway access [8]. In advanced cases, airway management becomes particularly difficult as cervical spine deformities reduce the range of motion required for conventional intubation techniques, necessitating alternative approaches. Techniques like fiberoptic instrument aided intubation are often recommended for airway management in these patients, but even these advanced techniques can fail. In extreme cases, alternative methods like blind nasotracheal intubation may be necessary [9]. This technique involves inserting the endotracheal tube through the nostril and advancing it into the trachea without direct visualization, initially guided by listening for breath sounds to approximate placement, followed by confirmation with capnography or other reliable techniques.

Many of the severe complications associated with AS, including fractures and spine deformities associated with difficult airway management, could be mitigated through early diagnosis and appropriate management. Treatments aimed at controlling the inflammatory process, such as nonsteroidal anti-inflammatory drugs (NSAIDs) and biologic agents targeting tumor necrosis factor (TNF) or interleukin-17 (IL-17), can slow disease progression and reduce the risk of severe spinal deformities. Physical therapy and postural training are also key components of long-term management, helping to maintain spinal flexibility and reduce deformity [1]. Early intervention may prevent the development of rigid deformities that complicate both trauma management and airway access, improving overall outcomes for patients with AS.

## Case presentation

**Figure 1:**
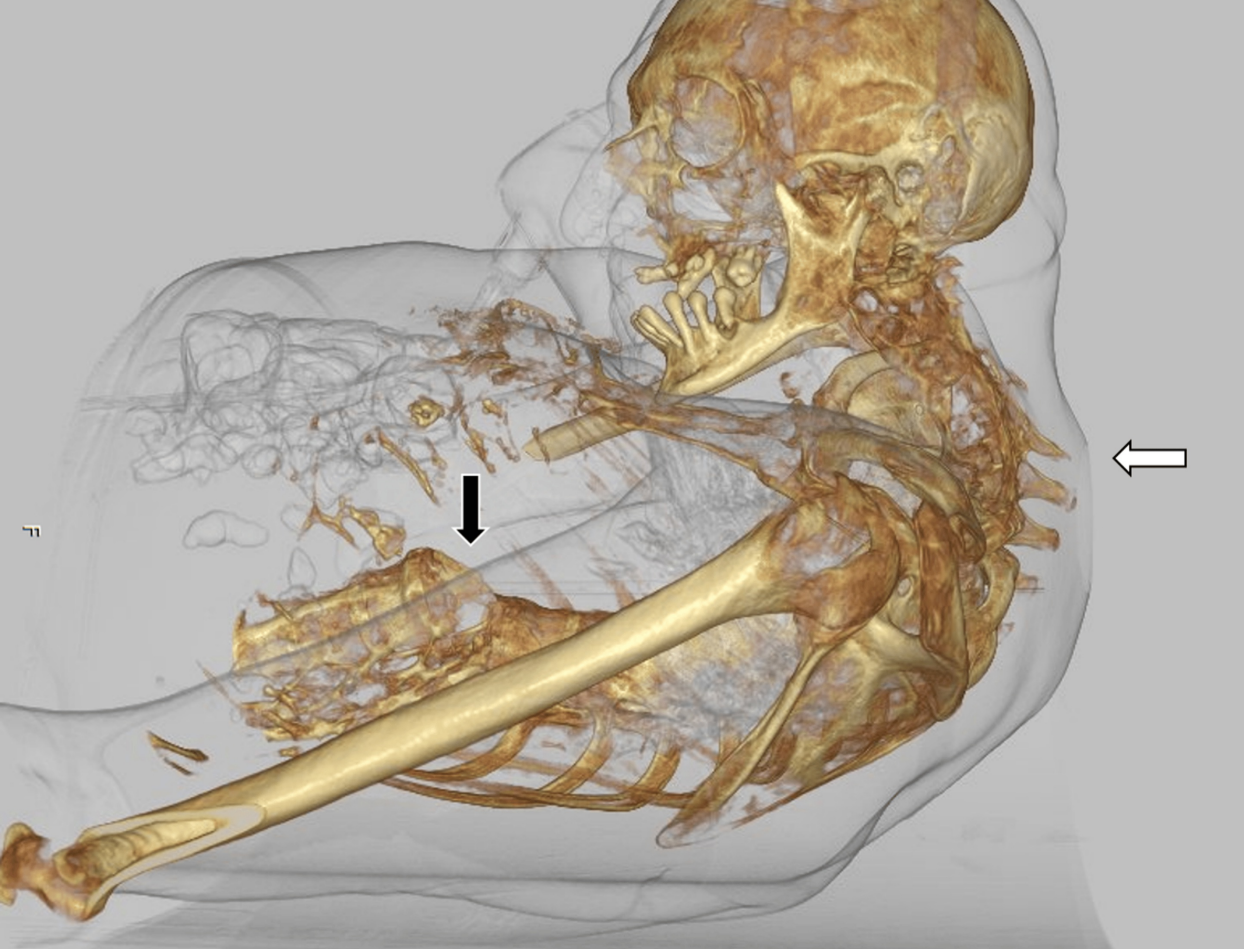
A 3D reconstruction of a CT scan revealing pronounced cervical deformities, characterized by severe hyperkyphosis and ankylosis (indicated by the white arrow), along with a spinal fracture (denoted by the black arrow). CT - computerized tomography

**Figure 2:**
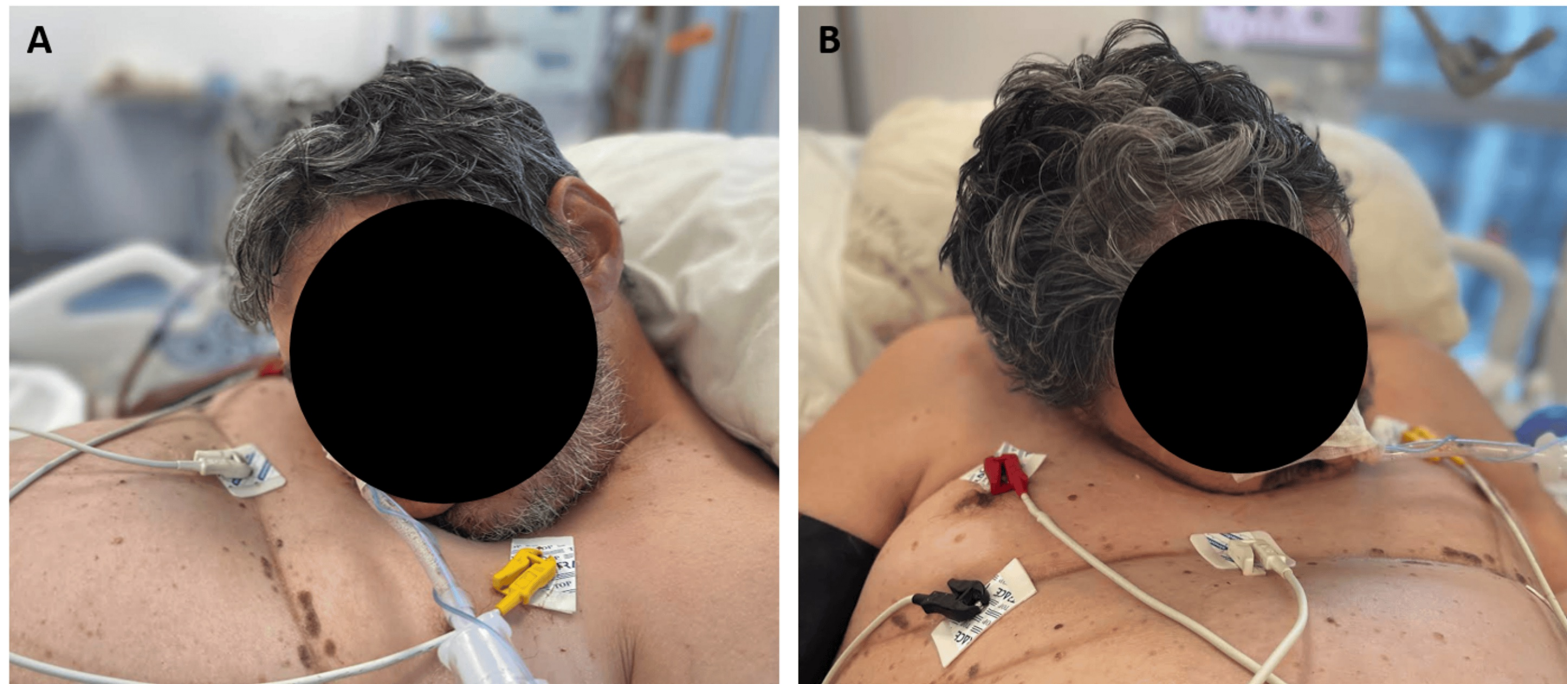
Severe cervical spine hyperkyphosis presenting as ''chin-on-chest'' deformity on (A) side and (B) front views.

A 48-year-old male with a history of untreated AS since the age of 19, presenting with severe cervical spine deformities (Figure 1, Figure 2), was admitted to the emergency department (ED) of a Level 1 trauma center following a fall from a one-meter height. His family history was notable for AS in his mother. He sustained no head injury but landed on his back and experienced immediate paraplegia. On arrival to the ED, the patient was alert and fully oriented with a Glasgow Coma Scale (GCS) score of 15. He presented with tachypnea and dyspnea, accompanied by hypotension with a blood pressure of 70/40 mm Hg and a heart rate of 80 beats per minute. He was breathing spontaneously, but oxygen saturation was low at 87% on room air. Neurological examination revealed a complete loss of motor and sensory function below the T5 level. Following fluid resuscitation and initial stabilization, the patient underwent diagnostic imaging. A computerized tomography (CT) scan of the chest and spine revealed bilateral rib fractures complicated by hemothorax, as well as unstable fractures of the T4 and L1 vertebral bodies, with associated spinal cord impingement. The patient was transferred to the intensive care unit (ICU) for ongoing stabilization prior to potential surgical intervention. Over the subsequent days, his condition deteriorated, leading to the development of acute respiratory failure, necessitating endotracheal intubation. However, due to the patient's significant cervical deformities, conventional orotracheal intubation guided by direct laryngoscopy was unsuccessful. Attempts at both video laryngoscopy and fiberoptic bronchoscope-assisted intubation also failed, as the conventional bronchoscope could not pass through either the mouth or the nose, and no ultrathin bronchoscope was available at the time. Both laryngeal mask airway intubation and Aintree catheter intubation attempts were unsuccessful as well. Surgical tracheostomy was deemed unfeasible due to the severity of the neck deformity. Ultimately, the patient was intubated endonasally in a semi-recumbent position, without transoral direct laryngoscopy and instrument-assisted tube advancement, using a blind technique. Tube placement was confirmed with positive capnography, chest X-ray and trans-tube bronchoscopy, and the patient was connected to mechanical ventilation. The patient was preoxygenated with 100% oxygen via a non-rebreather mask for five minutes prior to each intubation attempt. To provide sedation without compromising spontaneous respiration, intravenous dexmedetomidine (1 mcg/kg loading dose) and a small dose of ketamine (0.5 mg/kg) were administered. There were no complications during any of the attempts. Despite aggressive supportive care, the patient's condition continued to decline, and he passed away four days later due to complications of his injuries.

## Discussion

Airway management in patients with AS, especially those with severe cervical spine deformities, presents significant challenges. The characteristic spinal rigidity and kyphosis limit head and neck mobility, impairing alignment of the oral, pharyngeal, and laryngeal axes required for direct laryngoscopy [3]. In patients with AS, predicting difficult intubation is crucial due to the structural limitations caused by spinal deformities. Key predictors of difficult intubation include limited neck extension, reduced interincisor distance, shortened sternomental distance, and a high modified Mallampati score. These factors are significantly correlated with the Bath Ankylosing Spondylitis Metrology Index (BASMI), which measures spinal mobility, and show no correlation with the Bath Ankylosing Spondylitis Disease Activity Index (BASDAI), which assesses disease activity. This distinction reflects that intubation difficulties in AS patients are primarily due to physical limitations from spinal rigidity, rather than the level of inflammatory disease activity. Therefore, mechanical restrictions in spinal mobility are the key contributors to airway management challenges, underscoring the need for comprehensive physical assessments prior to intubation in AS patients [10]. 

Despite the challenges, there are alternative intubation strategies for AS patients that have been explored with varying success. Fiberoptic intubation remains the gold standard for patients with anticipated difficult airways, as it provides direct visualization with minimal manipulation of the cervical spine. A case series involving 12 patients with AS and difficult airways demonstrated that fiberoptic nasotracheal intubation in a semi-reclining position under conscious sedation was both safe and effective. The only reported side effect was minor epistaxis, which occurred in seven patients, indicating that this method is generally well-tolerated [11]. Another case series involving 20 patients with AS, 11 of whom had difficult airways, demonstrated that nasotracheal intubation under video laryngoscopy guidance was superior to classic direct laryngoscopy. The video-guided method was successful in eight out of the 11 patients with difficult airways, highlighting its effectiveness in managing complex cases [12]. Another alternative is the intubating laryngeal mask airway (LMA), which bypasses the need for direct visualization of the vocal cords, providing an airway conduit that can be used to pass an endotracheal tube. A study involving 11 patients demonstrated that LMA intubation was successfully performed on the first attempt in all cases, suggesting it as a viable option for airway management in patients with severe AS, both in elective and emergency situations [13]. The Aintree intubation catheter has also been successfully utilized for airway management in patients with severe AS, proving effective in managing difficult airways [14]. However, in certain extreme cases, including the one presented in this paper, even advanced techniques may fail. The failure of more modern techniques may be attributed to severe anatomical restrictions caused by extensive cervical kyphosis. According to the 2022 American Society of Anesthesiologists’ (ASA) Practice Guidelines for Management of the Difficult Airway, tracheostomy is regarded as a last-resort option when all other airway management techniques have failed. This approach is typically reserved for cases where noninvasive or less invasive methods are ineffective in securing the airway. However, the associated risks, such as complications from the procedure itself and potential infection, make tracheostomy a less favorable choice unless absolutely necessary [15]. Even so, although considered, tracheostomy was deemed unfeasible in our case due to the patient's deformity, which fixed the neck to the chest, rendering the anterior neck inaccessible for the procedure. With both traditional and advanced airway management strategies proving ineffective due to the severity of the cervical deformity, blind nasal intubation became the only viable option. While less commonly used in contemporary practice, blind nasal intubation can be a lifesaving procedure in difficult airway cases where other techniques are unsuccessful. Its success relies on anatomical landmarks and patient positioning rather than visual confirmation of the tube's passage through the vocal cords. This method, while effective in some cases, carries risks such as nasal trauma, bleeding, and potential tube misplacement, making it a high-risk procedure when no other alternatives are available [9].

## Conclusions

Airway management in AS patients with severe cervical deformities remains a complex and high-risk procedure. While techniques such as fiberoptic intubation and video laryngoscopy provide valuable tools for managing these airways, more traditional methods like blind nasal intubation still have a role in extreme cases. This case highlights the critical importance of early and effective treatment to prevent the development of airway-compromising deformities in patients with AS and underscores the need for a flexible, multidisciplinary approach to managing the airway in patients with advanced AS. Furthermore, it emphasizes how crucial it is to assess the airway in all AS patients proactively. Timely evaluation ensures that the most appropriate approach can be planned if airway management becomes necessary. This assessment is essential to improving patient outcomes and reducing complications in airway management. Moreover, it is essential to include training in blind nasotracheal intubation for personnel involved in advanced airway management, as this technique, though often considered outdated, remains a crucial option in select cases where conventional methods may fail.
